# Emotion Recognition from Single-Trial EEG Based on Kernel Fisher's Emotion Pattern and Imbalanced Quasiconformal Kernel Support Vector Machine

**DOI:** 10.3390/s140813361

**Published:** 2014-07-24

**Authors:** Yi-Hung Liu, Chien-Te Wu, Wei-Teng Cheng, Yu-Tsung Hsiao, Po-Ming Chen, Jyh-Tong Teng

**Affiliations:** 1 Department of Mechanical Engineering, Chung Yuan Christian University, Chungli 32023, Taiwan; E-Mails: eric.cheng.w@gmail.com (W.-T.C.); chariestas520@gmail.com (Y.-T.H); momobeer@livemail.tw (P.-M.C.); jtteng1@gmail.com (J.-T.T.); 2 School of Occupational Therapy, College of Medicine, National Taiwan University, Taipei 10051, Taiwan; E-Mail: chientevincewu@gmail.com

**Keywords:** EEG, emotion recognition, health care, brain-computer interface, support vector machine

## Abstract

Electroencephalogram-based emotion recognition (EEG-ER) has received increasing attention in the fields of health care, affective computing, and brain-computer interface (BCI). However, satisfactory ER performance within a bi-dimensional and non-discrete emotional space using single-trial EEG data remains a challenging task. To address this issue, we propose a three-layer scheme for single-trial EEG-ER. In the first layer, a set of spectral powers of different EEG frequency bands are extracted from multi-channel single-trial EEG signals. In the second layer, the kernel Fisher's discriminant analysis method is applied to further extract features with better discrimination ability from the EEG spectral powers. The feature vector produced by layer 2 is called a kernel Fisher's emotion pattern (KFEP), and is sent into layer 3 for further classification where the proposed imbalanced quasiconformal kernel support vector machine (IQK-SVM) serves as the emotion classifier. The outputs of the three layer EEG-ER system include labels of emotional valence and arousal. Furthermore, to collect effective training and testing datasets for the current EEG-ER system, we also use an emotion-induction paradigm in which a set of pictures selected from the International Affective Picture System (IAPS) are employed as emotion induction stimuli. The performance of the proposed three-layer solution is compared with that of other EEG spectral power-based features and emotion classifiers. Results on 10 healthy participants indicate that the proposed KFEP feature performs better than other spectral power features, and IQK-SVM outperforms traditional SVM in terms of the EEG-ER accuracy. Our findings also show that the proposed EEG-ER scheme achieves the highest classification accuracies of valence (82.68%) and arousal (84.79%) among all testing methods.

## 1. Introduction

Emotion recognition (ER) has recently attracted increasing attention in the fields of affective computing and human–computer interface (HCI), because ER is a key component spanning a variety of useful applications where emotion monitoring is required. Examples include human-like HCI systems [1] and healthcare [2]. A human-like HCI system should be equipped with a subset of human emotional skills, so as to interact with users in a more human-like and effective manner [1]. In healthcare applications, ER can improve quality of life for a wide spectrum of users (e.g., elderly people, chronically ill people, and patients with depressive disorders or severe motor disabilities) by assessing their emotional states and providing them with prompt and appropriate feedbacks or medical treatment suggestions [2].

### 1.1. Background

Depending on the signal sources, the existing ER methods can be divided into three categories. The first category of ER relies on visual signals of facial expression [3] and auditory signals of speech [4]. The second category of ER relies on multiple physiological signals generated by autonomic nervous system (ANS), such as skin conductance, electrocardiogram (ECG), and blood pressures. The first successful study in this category can be traced back to the method of Picard *et al.* [5] developed at the M.I.T. Media Lab. However, since Davidson and Fox [6] found that the electroencephalogram (EEG) signal generated from the central nervous system (CNS) could discriminate between positive and negative emotions in early 1980s, researchers started to consider the possibility of using brain-derived signals such as electroencephalograms (EEGs) as the basis of emotion recognition, which forms the third category of ER. In this category, emotions are often induced by using stimuli, such as International Affective Digitized Sounds (IADS) [7], and Bouchard's synthesized musical clips [8].

Evidence from contemporary neuroscience has shown that various cortical and subcortical regions (e.g., insula, prefrontal regions, thalamus, amygdala, hippocampus, basal ganglia) are involved in emotional perception and regulation [9–11]. In particular, the orbitofrontal cortex, a small region located in the ventral prefrontal cortex, is not only involved in emotional perception [9] but also involved in decision making [12]. Thus, it is globally agreed that emotions play an important role in decision making and human interaction [13]. Due to the partially localized nature of brain function for the emotional processes, EEG data that reflects summed brain activities of neuronal ensembles appears to be a plausible approach in emotion recognition. Recently, an increasing number of works that deal with various EEG-based ER (EEG-ER) problems have been presented, e.g., [14–29].

### 1.2. Related Works

**Figure 1.**
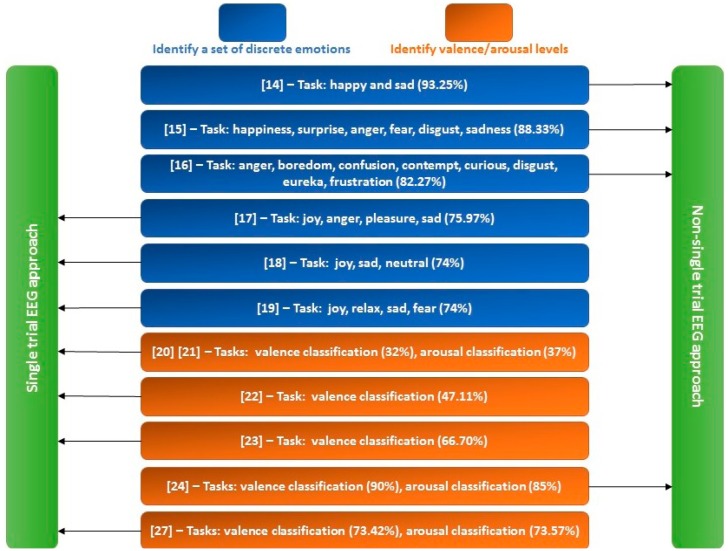
Comparison of related EEG-ER works.

Input to an EEG-ER system can be either single trial or non-single trial EEG signals. For a single-trial EEG-ER system, the input is the EEG signal recorded during one single trial [17–23,27,28], while the input for a non-single trial EEG-ER system is the averaged EEG signals of multiple trials [14–16,24]. For instance, the input of the method in [27] is raw EEG signals of 7-s in duration acquired during one trial, while the input to the EEG-ER system in [24] is the averaged 2.5-s EEG epochs collected from 40 trials. The signal averaging process can greatly improve the signal-to-noise ratio (SNR) of EEG signals. Therefore, the non-single trial approach is generally capable of achieving higher ER accuracy (see Figure 1 for a comparison) and is more suitable for establishing subject-independent ER systems. However, a large number of EEG epochs is in general required for a non-single trial system to perform the signal averaging process, which results in a time-consuming EEG recording procedure. In contrast, the single-trial approach is more suitable for EEG-ER applications where immediate feedback is required, such as human-robot interactions and real-time emotion monitoring. For example, a robot needs to identify the emotional state of a person in a few seconds in order to provide a prompt and appropriate response to the person. Another potential application lies in assuring flight/driving safety in which arousal level of a pilot/driver need to be persistently monitored: a pilot/driver needs to be alerted when he/she is tired.

EEG-ER systems can be divided into two major types of emotional classification: one is based on the discrete emotional model [29] and the other is based on the bi-dimensional emotional model [30]. The two models have been widely adopted for operationally defining the scope of emotions. Ekman [29] has suggested six basic emotions: happiness, surprise, fear, anger, sadness, disgust, and other emotions such as disappointment and shame are spanned by the six basic emotional states. Previous EEG-ER systems adopting this discrete emotional model were designed to classify a set of discrete emotions [14–19]. While these discrete emotional labels are easy to understand, some of the discrete emotion labels do not have exact translations across different language. For example, “disgust” does not have an exact translation in Polish [31]. Therefore, psychologists prefer the bi-dimensional emotional model which categorizes emotions into a two-dimensional space spanned by two affective variables, valence and arousal [32,33], where valence ranges from unpleasant to pleasant and arousal ranges from passive or calm to active or excited. Accordingly, emotions can be further classified as one of the four emotional categories in the valence-arousal space: high valence and high arousal (HVHA), low valence and high arousal (LVHA), low valence and low arousal (LVLA), and high valence and low arousal (HVLA). Previous methods following this model were mainly focused on two binary classification problems [20–24,27,28]: valance (high valance *vs.* low valance) and arousal (high arousal *vs.* low arousal) classifications. Although the two emotional models define emotions with different perspectives, there is a connection between the two models. For instance, “happy” and “sadness” belong to the HVHA and LVLA emotional categories, respectively. Also, both “anger” and “disgust” are in the same category of LVHA because they are all negative and highly arousing emotions.

Although there are quite a few factors that can influence the EEG-ER accuracy (e.g., participants' experience, type of emotional stimuli, number of electrodes and their locations, EEG representation and classification methods), the results of previous EEG-ER methods shown in Figure 1 indeed demonstrate that: (1) non-single trial approach performs better than single trial approach, and (2) the accuracies of valence and arousal classifications are much lower than those of discrete emotion classifications on average. These up-to-date reviews therefore indicate the fact that valence and arousal classifications based on single-trial EEG signal are still challenging issues to be addressed.

### 1.3. Presented Work

To address this issue, we propose in this paper a three-layer EEG-ER scheme. The input to this framework is the single-trial multi-channel EEG, and its outputs include the labels of valence and arousal. In the first layer, EEG signal of each channel is first bandpass-filtered into multiple frequency bands, and then the spectral powers of these frequency bands are calculated. The second layer further computes, and then concatenates the multiple band powers from different channels into one single power-spectral vector. This vector is then sent into the second layer for discriminating feature extraction and dimensionality reduction. The kernel Fisher's discriminant analysis (KFDA) [34] is employed to accomplish this task. The feature vector extracted by KFDA is called a kernel Fisher's emotion pattern (KFEP) in this paper for it can capture useful information in discriminating emotions. The third layer starts to perform valence and arousal classifications as soon as it receives the KFEP from the second layer. Most EEG-ER methods adopted the support vector machine (SVM) [35,36] as the emotion classifier due to its success in various pattern recognition applications. However, EEG-ER suffers from the problem of imbalanced training datasets, and this problem would tend to degrade the generalization performance of SVM. To deal with this problem, the imbalanced SVM [37,38] is employed as the basis of emotion classifier in this paper. Moreover, the quasiconformal kernel transformation technique of Wu and Amari [39] is applied to further improve the generalization performance of the imbalanced SVM. By incorporating the DEC algorithm and the quasiconformal kernel transformation technique into SVM, an imbalanced quasiconformal kernel SVM (IQK-SVM) is proposed and serves as the emotion classifier of the third layer. In Section 2, the problem of imbalanced EEG dataset and the proposed ideas are described in more details. Then, the KFEP and IQK-SVM will be formulated in Section 3.

In addition to the EEG-ER method, we also modified an emotion induction paradigm [21] to collect representative EEG data of different emotional states from participants. In order to effectively induce designated emotions, a set of pictures selected from the International Affective Picture System (IAPS) [40] are employed as the emotion induction stimuli. More details about the emotion induction paradigm and corresponding results will be discussed in Section 4.

## 2. Problem Descriptions and Solutions

### 2.1. Emotion Classifier Design

Emotion classifier plays a critical role in an EEG-ER system for it primarily determines the generalization performance of the system. Most of previous studies have used SVM as the emotion classifier in their EEG-ER systems [14,15,17,19–24,32,33]. However, the performance of SVM tends to decline due to the problem of imbalanced emotional datasets described as follows:

#### Problem description

In EEG-ER study, an emotion-induction procedure consisting of a number of trials is necessary in order to collect emotional EEG datasets used for training and testing the classifier. Specifically, it is critical to keep the numbers of stimuli equal across different emotion classes (e.g., the same numbers of high-arousal stimuli and low-arousal stimuli will be presented to a participant) [15,21,24,27]. Each trial typically consists of a stimulus-presentation phase and a self-assessment phase. During the stimulus-presentation phase, an emotional stimulus is presented to induce an expected emotion for a participant, where the expected emotion denotes the emotion that the stimulus is supposed to evoke. After the completion of this phase, the participant proceeds to a self-assessment phase in which the participant assesses the emotion that he/she experienced during the corresponding stimulus-presentation phase through computerized tools such as the self-assessment manikin (SAM) [41]. The self-assessment result is then used to label the EEG data recorded during the stimulus-presentation phase. However, due to the subjectivity of emotion, it is possible that the experienced emotion differs from the expected one [15,20]. For instance, a participant may experience an intensive (*i.e.*, high arousal) emotion even if the stimulus presented to the participant is “supposed to be” emotionally non-intensive [32]. In other words, the emotional EEG dataset collected during the entire emotion-induction procedure is most likely imbalanced: high-arousal EEG data outnumber or are outnumbered by low-arousal ones, even if the number of high-arousal stimuli equals to the number of low-arousal ones. In SVM, one of the objectives during training phase is to minimize the number of training errors from both classes. A training error occurs when the classifier mistakenly classify an EEG segment as belonging to an emotional category which is different from the emotional labeling based on the participant's self-assessment (e.g., when a participant report experiencing an emotional state that belongs to HVHA but the classifier categorize the corresponding EEG segment as belonging to HVLA). Due to the fact that the penalty weights for both classes are the same in SVM, the optimal separating hyperplane (OSH) trained by the SVM is skewed toward the minority class (*i.e.*, the class with fewer training samples), known as the class-boundary-skew problem [37]. As a result, applying SVM to classify an imbalanced emotional dataset may lead to unsatisfactory emotion classification accuracy.

#### Existing solutions

There have been various methods presented to address the problem of learning from imbalanced datasets where one class (majority class) is larger than the other (minority class). A nice survey on these methods have been provided in [42,43]. These methods are based on either data preprocessing or SVM approaches, briefly introduced as follows:

(1) *Data preprocessing approach*. Methods in this category are based on either oversampling or undersampling techniques. Random oversampling adds data sampled from the minority class to the minority-class dataset. Random undersampling removes data from the majority-class dataset. The two techniques are functionally equivalent, because they both alter the size of a dataset to balance the class distributions within the dataset. However, undersampling may result in an inherent loss of valuable information [44,45]. On the other hand, oversampling may result in overfitting [46]. Cluster-based oversampling [47], synthetic minority oversampling technique (SMOTE) [48], and its variant—ADASYN [49] are proposed to correct this drawback. However, oversampling increases the number of training data, which greatly lowers the training speed of SVM because SVM is computationally expensive [50]: its training time complexity is *O*(*L*^3^), where *L* is the size of the training set. This drawback further magnifies when a cross validation procedure is performed to optimize the SVM, which would reduce the usability of an EEG-ER system in practical use since the training speed of a system is one of the crucial factors that should be considered.
(2) *SVM approach*. Methods in this category solve the class imbalance problem by directly modifying the formulation of the regular SVM. The most typical example is the DEC algorithm [37], which assigns a heavier penalty to the minority class and a slighter penalty to the majority class during the training phase. By doing so, the class-boundary-skew problem of SVM can be solved because the learned OSH can be more distant from the minority class. The imbalanced SVM [37], the SVM with output-moderation ability [51], and the TAF-SVM [38] are developed based on the DEC algorithm.

#### Proposed emotion classifier

In this study, the imbalanced SVM is adopted to prevent the class-boundary-skew problem due to imbalanced emotional EEG datasets. Moreover, we also introduce quasiconformal kernel transformation [38] technique into the imbalanced SVM so that its generalization performance can be further improved. The quasiconformal kernel transformation [39] was originally designed for SVM and the basic idea is to enlarge the spatial resolution around the OSH of SVM in a kernel-induced feature space by quasiconformally transforming a preselected kernel (e.g., polynomial or RBF kernels). Furthermore, enlarging the spatial resolution around the OSH is equivalent to increasing the separability of classes. Since the separation margin between classes is increased, the generalization performance of SVM is improved. This kernel transformation technique has also been applied to other classifiers like *k*-nearest neighbor (*k*-NN) method [52] and support vector data description (SVDD) [53]. Motivated by their success, we propose in this paper a novel emotion classifier, the imbalanced quasiconformal kernel SVM (IQK-SVM), by introducing this kernel transformation technique into the imbalanced SVM. The IQK-SVM is able to achieve higher classification accuracies of valence and arousal than the commonly used SVM emotion classifier, as will be shown in Section 4.

### 2.2. Feature Extraction

Feature selection also plays a critical role in designing an EEG-ER system. In previous EEG-ER systems, various features have been used, including common spatial pattern [14], higher order crossings [15], time-domain statistical features [15,19], EEG spectral power [8,17,19–21,24,25,33], wavelet entropy [26], and coherence feature [28]. Among these features, EEG spectral power appears to be the most frequently used feature, however, two limitations should be noted. First, with the increases of the number of electrodes and the number of chosen frequency bands, the dimension of the feature vector consisting of the spectral powers from different bands and different electrodes would be very large which increase the computational burden. Second, EEG suffers from the problem of volume conduction effect [54] so that the EEG spectral powers extracted from the surface EEG signals might not be able to well represent the underlying oscillatory brain activity during emotional processing. Therefore, it is necessary to use a post-processing method to further extract fewer features with higher discriminability from a large number of spectral powers of different bands and electrodes. This can be achieved by using subspace analysis methods, such as principal component analysis (PCA) and linear discriminant analysis (LDA).

Recently, we have used the nonlinear version of PCA, the kernel PCA [55], to extract features from the spectral powers, and the extracted features form a new feature vector called kernel eigen-emotion pattern (KEEP) [27]. Our preliminary results reported in [27] have demonstrated that the features extracted by kernel PCA (*i.e.*, the KEEPs) can give higher classification accuracies of both valence and arousal than the features extracted by PCA. This should be attributed to the fact that kernel PCA can compute higher-order statistics (*i.e.*, nonlinear dependency) among three or more EEG spectral powers from different bands and channels while PCA can only compute the second-order correlation (*i.e.*, linear dependency) of two spectral powers. In other words, kernel PCA can better capture the patterns of complex brain activity during emotional processing.

In this study, we adopt another powerful kernel method, the kernel Fisher's discriminant analysis (KFDA) [34], as the dimensionality reduction tool. KFDA is a nonlinear version of LDA. It first maps data into a higher dimensional space through a nonlinear mapping and then performs LDA in that space to maximize the between-class scatter and minimize the within-class scatter of the mapped data with a supervised learning manner. Since the objective of KFDA is to generate a set of new features that result in the maximized class separability, the features by KFDA would have better discrimination ability in pattern classification than the ones by kernel PCA because kernel PCA merely diagonalizes covariance of mapped data in an unsupervised learning manner: diagonalizing data covariance does not guarantee a new feature set with a large class separability. KFDA has been applied to various pattern recognition problems where feature extraction and dimensionality reduction are required, such as face [56] and electromyography (EMG) [57] recognition. In this paper, we combine the frequently-used EEG spectral power feature and the KFDA to derive a new feature called kernel Fisher's emotion pattern (KFEP).

In summary, we propose a three-layer EEG-ER scheme that integrates a novel emotion classifier IQK-SVM and a new EEG feature extraction method KFEP. In the following, we provide an overview of the scheme and then formulate KFEP and IQK-SVM.

## 3. Methods

### 3.1. Overview of the Proposed Three-Layer EEG-ER Scheme

**Figure 2.**
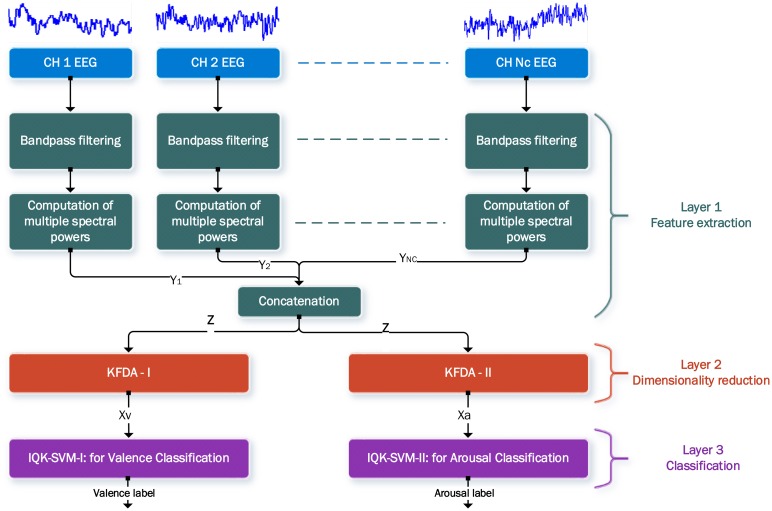
Proposed kernel machine-based three-layer EEG-ER scheme. In this figure, **z** denotes a spectral power (SP) vector, and both Xa and Xv are KFEP.

The proposed EEG-ER scheme is illustrated in Figure 2. This scheme consists of three layers. The first layer performs feature extraction. For the current study, five EEG frequency bands are selected as the representative event-related oscillation during emotional processing, including theta (4–8 Hz), alpha (8–13 Hz), low beta (13–20 Hz), high beta (20–30 Hz), and gamma (30–45 Hz) bands.

Therefore, a set of fifth-order Butterworth band-pass filters is conducted to span the five indicated bands. Then for the EEG signal of each channel, power of each frequency band is calculated by squaring and averaging the samples in the filtered signal. The spectral power features extracted from one single channel are concatenated to a 5-dimensional vector **y**. The vectors from all the channels are further concatenated to generate a spectral-power vector **z** of *n* dimension, where *n* = *N_c_* × 5 and *N_c_* is the number of channels.

In layer 2, the dimensionality reduction is performed in a parallel way: the same spectral power vector inputs into two different KFDA components, KFDA-I and KFDA-II. Here, the term “different” means that their optimal parameters (including the number of chosen eigenvector and the kernel parameter) are different after an off-line training process: KFDA-I is trained with high-valence and low-valence EEG data while KFDA-II is trained with high-arousal and low-arousal data. The vectors generated by KFDA-I and KFDA-II are KFEPs in this paper, and are fed into the emotion classifiers IQK-SVM-I and IQK-SVM-II for classification, respectively. IQK-SVM-I is responsible for valence classification and IQK-SVM-II performs the arousal classification, and they are trained independently. Finally, kernel machine-based three-layer EEG-ER scheme generates two classification outputs, including the valence label (high or low) and arousal label (high or low).

To facilitate the following, high-valence emotion and low-valence emotion are defined as positive and negative classes, respectively. Similarly, high-arousal and low-arousal emotions are also defined as positive and negative classes, respectively. Mathematical symbols representing vectors and matrices are all expressed in boldface throughout this paper.

### 3.2. Generate KFEP by KFDA

Here the EEG spectral power vectors are simply called data. Given a set of positive and negative training data **z***_i_* ∈ *R^n^*,*i* = 1, …,*L*, KFDA first maps the data into a higher dimensional feature *F via* a nonlinear mapping *φ*:**z** ∈ *R^n^* → *φ* (**z**) ∈ *F*, and then solves the following maximization problem:

(1) $$\underset{v}{\text{Maximize}}\;\frac{{\text{v}}^{t}\text{Bv}}{{\text{v}}^{t}\text{Wv}}$$

where the two matrices:

(2) $$\text{W}=\frac{1}{L}\sum_{p=1q=1}^{2}\sum_{q=1}^{n_{p}}\phi \left({\text{z}}_{pq}\right)\phi^{T}\left({\text{z}}_{pq}\right)\;\text{and}\;\text{B}=\frac{1}{L}\sum_{p=1}^{2}n_{p}\left(\frac{1}{n_{p}}\sum_{q=1}^{n_{p}}\phi \left({\text{z}}_{pq}\right)\right){\left(\frac{1}{n_{p}}\sum_{r=1}^{n_{p}}\phi \left({\text{z}}_{pr}\right)\right)}^{t}$$

represent the within-class and the between-class scatters in *F*, respectively, ***z****_pq_* denotes the *q*th training data in the *p*th class, *n_p_* is the number of training data in the *p*th class, and *t* denotes the transpose of a matrix. Note that the mapped data are centered to have zero mean in *F* (see Appendix C of [34] for a derivation of the centering method). Solving the optimization problem in Equation (1) is equivalent to solving the eigenvalue problem:

(3) λWv=Bv

The projection vector **v** is the eigenvector of **W**^−1^**B** and is spanned by the mapped training data: $\text{v}=\sum_{i=1}^{L}a_{i}\phi \left({\text{z}}_{i}\right)$, where *a_i_* are the expansion coefficients for **v**. By using the kernel function *K*(***z****_i_*,***z****_j_*) = *K_ij_* ≡ *φ*(**z***_i_*)·*φ*(**z***_j_*) and performing the eigenvector decomposition on the *L* × *L* kernel matrix **K**=(*K_ij_*), we get *L* normalized expansion coefficients for each projection vector: $\text{a}=\text{a}/\sqrt{{\text{a}}^{t}\text{Ka}}$, where **a** is the eigenvector of **K**. The projection of a test data **z** onto the *i*th projection vector **v***_i_* is computed by:

(4) $$x^{i}={\text{v}}^{i}‥\phi \left(\text{z}\right)=\sum_{j=1}^{L}a_{j}^{i}\phi \left({\text{z}}_{j}\right)‥\phi \left(z\right)=\sum_{j=1}^{L}a_{j}^{i}K\left({\text{z}}_{j},\text{z}\right),i=1,\ldots ,L$$

where $a_{j}^{i}$ denotes the *j*th expansion coefficient of the *i*th projection eigenvector. By choosing the first *d* projection values, a *d*-dimensional vector **x** = (*x*^1^,…,*x^d^*)*^t^* ∈ *R^d^* is obtained, which is a KFEP.

In this paper, the Gaussian kernel:

(5) $$K\left({\text{z}}_{i},{\text{z}}_{j}\right)=\exp\left(-{\left\|{\text{z}}_{i}-{\text{z}}_{j}\right\|}^{2}/2\sigma^{2}\right)$$

is adopted, where *σ* is the kernel parameter. Therefore, the free parameters of KFDA include *d* and *σ*, which are experimentally determined and can be optimized by a cross-validation procedure.

### 3.3. Classification of KFEP via IQK-SVM

In this subsection, we first formulate the imbalanced SVM and then review the basics of the quasiconformal kernel transformation. Finally, we summarize the proposed imbalanced quasiconformal kernel SVM (IQK-SVM)

#### 3.3.1. Imbalanced SVM

Here, the KFEPs are simply called data. Given a set of training data $S={\left\{{\text{x}}_{i}\in R^{d},y_{i}\right\}}_{i=1}^{L}$, where **x***_i_* are training data, *y_i_* ∈{ −1,+1} are class labels, imbalanced SVM maps the training data into a higher-dimensional feature space via *φ* and then finds the OSH: *H* = **w***_t_ φ* (**x**)+*b*=0 that maximizes the margin of separation and minimizes the training error simultaneously. Let *I*_+_ = {*i*|*y_i_* = +1} and *I*_−_ = {*i*|*y_i_* = −1}. Imbalanced SVM which is formulated as:

(6) $$\begin{array}{ll} \text{Minimize} & \frac{1}{2}{\left\|\text{w}\right\|}^{2}+C^{+}\sum_{i\in I_{+}}\xi_{i}+C^{-}\sum_{i\in I_{-}}\xi_{i}\\ \text{subject}\;\text{to} & \begin{array}{cc} y_{i}\left(\text{w}‥\phi \left({\text{x}}_{i}\right)+b\right)-1+\xi_{i}\geq 0, & i=1,\ldots ,L \end{array}\\ & \begin{array}{cc} \xi_{i}\geq 0, & i=1,\ldots ,L \end{array} \end{array}$$

where *ξ_i_* are slack variables representing training errors, and *C*^+^ and *C*^−^ are penalty weights for positive class and negative class, respectively. Introducing the Lagrangian to Equation (6) yields the dual problem:

(7) $$\begin{array}{ll} \text{Maximize} & \sum_{i=1}^{L}\alpha_{i}-\frac{1}{2}\sum_{i=1}^{L}\sum_{j=1}^{L}\alpha_{i}\alpha_{j}y_{i}y_{j}K\left({\text{x}}_{i},{\text{x}}_{j}\right)\\ \text{subject}\;\text{to} & 0\leq \alpha_{i}\leq C^{+},\forall i\in I_{+},\\ & 0\leq \alpha_{i}\leq C^{-},\forall i\in I_{-},\\ & \sum_{i=1}^{L}\alpha_{i}y_{i}=0, \end{array}$$

where *α_i_* are Lagrange multipliers. The training data points with 0 < *α_i_* ≤ *C*^+^ or 0 < *α_i_* ≤ *C*^−^ are called support vectors (SVs). The weight vector of OSH is given by:

(8) $$\text{w}=\sum_{x_{i}\in SV}\alpha_{i}y_{i}\phi \left({\text{x}}_{i}\right).$$

The optimal value of *b* can be determined by taking any training data point whose 0 < *α_i_* < *C*^+^ or 0 < *α_i_* < *C*^−^ into the Kuhn-Tucker (KT) conditions:

(9) $$\begin{array}{l} \alpha_{i}\left[y_{i}\left(\text{w}‥\phi \left({\text{x}}_{i}\right)+b\right)-1+\xi_{i}\right]=0,i=1,\ldots ,L\\ \left(C^{+}-\alpha_{i}\right)\xi_{i}=0\;\text{for}\;y_{i}=+1,\\ \left(C^{-}-\alpha_{i}\right)\xi_{i}=0\;\text{for}\;y_{i}=-1. \end{array}$$

The class label for a test data can be obtained using the decision function:

(10) $$D\left(\text{x}\right)=\sum_{{\text{x}}_{i}\in SV}\alpha_{i}y_{i}K\left({\text{x}}_{i},\text{x}\right)+b.$$

The test data is classified as positive if *D*(**x**) > 0; negative otherwise. The three following remarks can be made:

(1) Based on the idea of DEC algorithm, it is clear that if positive class is the minority class (the class with fewer training data), then we need to set *C*^+^ > *C*^−^ to prevent the class-boundary-skew problem caused by the imbalanced training set.
(2) On the contrary, the setting of *C*^+^ < *C*^−^ is required if the positive class is the majority one.
(3) If *C*^+^ < *C*^−^, the imbalanced SVM becomes the conventional SVM, which is formulated as follows:
  $$\begin{array}{ll} \text{Maximize} & \frac{1}{2}{\left\|\text{w}\right\|}^{2}+C\sum_{i=1}^{L}\xi_{i}\\ \text{subject}\;\text{to} & \begin{array}{cc} y_{i}\left(\text{w}‥\phi \left({\text{x}}_{i}\right)+b\right)-1+\xi_{i}\geq 0, & i=1,\ldots ,L \end{array}\\ & \begin{array}{cc} \xi_{i}\geq 0, & i=1,\ldots ,L. \end{array} \end{array}$$

According to the SVM formulation, we can see that if the two classes are completely separable in the space of EEG patterns, there will be no training errors. As a result, the aforementioned class-boundary-skew problem will not occur even if the two classes are severe imbalanced.

#### 3.3.2. Basics of Quasiconformal Kernel Transformation

It is known that the nonlinear mapping *φ* associated with the Gaussian kernel embeds the input space *R^d^* into a feature space *F* of infinite dimension as a Riemannian manifold [39]. The Riemannian metric induced by the kernel is given by:

(11) $${g_{ij}\left(\text{x}\right)={\left(\frac{\partial \varphi \left(x\right)}{\partial x_{i}}\right)}^{T}\left(\frac{\partial \varphi \left(\text{x}\right)}{\partial x_{j}}\right)=\frac{\partial^{2}K\left(\text{x},{\text{x}}^{\prime }\right)}{\partial x_{i}\partial x_{j}^{\prime }}|}_{{\text{x}}^{\prime }=\text{x}}$$

where *x_i_* is the *i*th component of the vector **x**. The Riemannian distance *ds* in the feature space caused by a small vector *d***x** is:

(12) $$ds^{2}=\sum_{i}\sum_{j}g_{ij}\left(\text{x}\right)dx_{i}dx_{j}$$

which shows how a local volume in *R^d^* is enlarged or contracted in *F* under the mapping of *φ*. A quasiconformal transformation of the Riemannian metric can be defined as:

(13) $${\tilde{g}}_{ij}(x)=\Omega (x)g_{ij}(x)$$

where Ω(**x**) is a scalar function of **x**. In practice, directly realizing this transformation is difficult because *φ* is in general unknown. Instead, we define a quasiconformal transformation of *φ* as follows:

(14) $$\tilde{\varphi }(x)=Q(x)\varphi (x)$$

where *Q*(**x**) is a positive real-valued quasiconformal function. Based on Equation (14), the quasiconformal transformation of the primary kernel *K* (*i.e.*, the Gaussian kernel in this paper) can be obtained as:

(15) $$\tilde{K}(x,x^{\prime })=Q(x)\phi (x)\cdot Q(x^{\prime })\phi (x^{\prime })=Q(x)Q(x^{\prime })K(x,x^{\prime }),$$

where *K̃* is a quasiconformal kernel (*i.e.*, the transformed kernel). Substituting Equation (15) back into Equation (9), we have the new metric *g̃_ij_*(**x**) associated with *K̃*:

(16) $${\tilde{g}}_{ij}(x)=(\partial Q(x)/\partial x_{i})(\partial Q(x)/\partial x_{j})+Q{\left(x\right)}^{2}g_{ij}(x)$$

If the quasiconformal function *Q*(**x**) is designed in a way that it has the largest value at the position of *φ*(**x**) and monotonically decreases with the distance from *φ*(**x**) in *F*, then *g̃_ij_*(**x**) will become larger around **x** and smaller elsewhere, as can be seen from Equation (16).

#### 3.3.3. IQK-SVM

In this study, we further improve the generalization performance of imbalanced SVM by the quasiconformal kernel transformation. The basic idea is as follow. The basic idea is as follows: first, if we would like to improve the generalization ability of imbalanced SVM, then we need to increase the separability of classes. Accordingly, the quasiconformal function *Q*(**x**) can be chosen in a way that it has the largest value at the position of the OSH and decays with the distance from the OSH in *F*. As such, the Riemannian metric around the preimage of the OSH in the original space of patterns (*i.e.*, the decision boundary in *R^d^*) is increased, as indicated in Equation (15). Since the Riemannian metric around the decision boundary is increased, it can be seen from Equation (11) that the volume of the area around the OSH is therefore magnified. Further, magnifying the volume of this area is equivalent to enlarging the spatial resolution around the OSH, which is equivalent to increasing the separability of classes.

The nonlinear mapping is unknown, and therefore the space *F* is implicit: the location of OSH (the hyperplane *H* = **w***_t_ φ* (**x**)+b = 0) in *F* is unknown. That is, the above idea is infeasible. However, if we define the set:

(17) $$T=\left\{{\text{x}}_{i}|{\text{x}}_{i}\in S\;\text{and}-1\leq D\left({\text{x}}_{i}\right)\leq 1\right\}$$

where *D* is the decision function expressed in (10), than it is clearly that the images of the data in the set *T* are exactly on the hyperplanes *H* = +1 or *H* = −1, or fall inside the regions between *H* = +1 and *H* = −1. In other words, the set *T* is actually a subset of the SVs of imbalanced SVM: *T* collects the SVs whose images falls inside the margin of separation between classes in *F*. We call the data points in *T* within-margin SVs. With set *T*, the idea aforementioned can thus be implemented in an indirectly manner. First, the function *Q*(**x**) is chosen in a way that it has the largest value at the position of the image of *T* and decays with the distance from *T* in *F*. Since the within-margin SVs are all inside the margin of separation, they are closed to the OSH. Therefore, increasing the spatial resolution around the image of *T* is equivalent to increasing the spatial resolution of the margin of separation in *F*. As a result, the class separability of imbalanced SVM is increased, and thus the generalization performance of imbalanced SVM is improved.

Accordingly, we choose the function *D*(**x**) to have larger values at the positions of the mapped SVs in *T* and smaller elsewhere. Similar to [53], the quasiconformal function *Q*(**x**) here is chosen as a set of Gaussian functions:

(18) $$Q\left(\text{x}\right)=\sum_{x_{i}\in T}\exp\left(-\frac{{\left\|\varphi \left(\text{x}\right)-\varphi \left({\text{x}}_{i}\right)\right\|}^{2}}{\tau_{i}^{2}}\right)$$

where the parameter *τ_i_* calculates the mean squared distance from *φ*(**x***_i_*) to its *N* nearest neighbors *φ*(**x***_k_*):

(19) $$\tau_{i}^{2}=\frac{1}{N}\sum_{k}{\left\|\varphi \left({\text{x}}_{k}\right)-\varphi \left({\text{x}}_{i}\right)\right\|}^{2}$$

It can be seen from Equation (18) that the function *D*(**x**) decreases exponentially with the distance to the images of the data belonging to the set *T*. In this study, we set *N*_= 3_. Finally, the training process of the proposed IQK-SVM is summarized as the two steps:

(1) Step 1: An imbalanced SVM is initially trained on a training set by a primary kernel.
(2) Step 2: Using the same training set, retrain the imbalanced SVM by the quasiconformal kernel defined in Equation (15).

## 4. Experimental Design, Results and Discussion

### 4.1. Emotional EEG Data Collection

#### 4.1.1. Participants and Experimental Protocol

Ten volunteers (19–24 y/o) with normal or corrected-to-normal vision participated in the current study. None of the participants had any neurological or psychological medical history. Before experiments, we obtained informed consent from each participant. The EEG recording equipment was connected with a personal computer. All emotional stimuli were presented on a 43.18 cm CRT screen and the participants were seated in a fixed and comfortable chair. The distance between the eyes of each participant and the CRT display was controlled to be about 60 cm. EEG signals were recorded by 65 Ag/AgCl electrodes mounted on an electro-cap (Qucik-Cap 64) from NeuroScan system (Compumedics Inc., Charlotte, NC, USA) where the locations of the electrodes follow the international 10–20 system. Among the 65 channels, two were references positioned at M1 and M1, and one was a Ground channel at forehead, which leads to a total of 62 EEG channels used in this study (*i.e.*, *N_c_* = 62). Therefore, when the spectral power features (theta, alpha, low beta, high beta, and gamma band powers) are used, the dimension of the feature vector *n* is 310 (n = *N_c_* × 5), as illustrated in Figure 2. Impedances of the electrodes were kept below 10 k Ω by applying electric gel. Ocular artifacts were monitored by horizontal (HEOR, HEOL) and vertical (VEOH, VEOL) bipolar EOG electrodes. Subsequently, the influence of EOG over the recorded EEG was removed by applying the artifact removal software of NeuroScan. Both EEG and EOG signals were subjected to a band-pass filtering (0.5–100 Hz) and digitized at a sampling frequency of 500 Hz using the NuAmps amplifier from NeuroScan Inc.

#### 4.1.2. Emotional Stimuli

In EEG-ER studies, it is critical to acquire representative EEG data that reflect different types of emotional states for training and testing an EEG-ER model. To achieve this goal, two emotion-induction approaches have been suggested [5]: one is the self-initiated emotion induction and the other is the event-triggered emotion induction. In the self-initiated emotion induction category, participants are instructed to recall past emotional episodes or to mimic the facial expression of specific emotional states (e.g., happiness and sadness). In the event-triggered emotion induction, one would use external emotional stimuli to induce designated emotion, and such stimuli are in general visual (e.g., pictures and video clips), auditory (e.g., music), or a combination of both. Popular standardized emotional stimuli repertoires include IAPS [39], IADS [7], and Bouchard's synthesized musical clips [8].

**Figure. 3.**
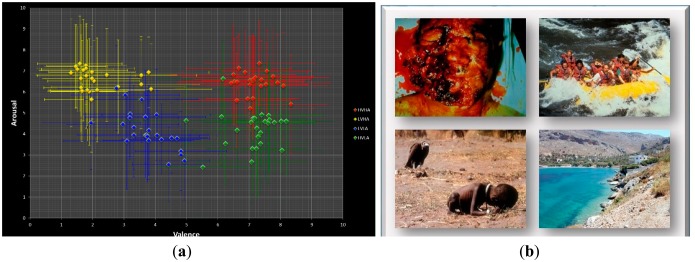
(**a**) Distribution of the selected 100 IAPS pictures in the valence-arousal plane, where each dot contains information of both the average score and the estimated standard deviations along the two axes and (**b**) examples of the selected pictures, where the upper-right, upper-left, bottom-left, and bottom-right pictures are HVHA, LVHA, LVLA, and HVLA stimuli, respectively.

IAPS pictures have been widely used as the emotional stimuli in previous EEG-ER studies [27,58]. All IAPS pictures were rated in terms of perceived valence and arousal levels (level 1–level 9). According to their valence and arousal scores, the IAPS pictures can be divided into four categories in the valence-arousal space: HVHA (first quadrant), LVHA (second quadrant), LVLA (third quadrant), and HVLA (fourth quadrant). An IAPS picture with a high arousal score (*i.e.*, arousal level > 5) represents an emotionally intensive stimulus while a picture with a low arousal score (*i.e.*, arousal level < 5) represents an emotionally calm stimulus. It is noticed that a HA picture can be any pictures belonging to the first or the fourth quadrants. On the other hand, an IAPS picture with a high valence score (*i.e.*, valence level > 5) represents a positive-emotion stimulus while a picture with a low valence score (*i.e.*, valence level < 5) represents a negative-emotion stimulus. However, the subjective emotional experiences and responses to a given stimulus are highly variate from person to person. In other words, the induced emotion is not necessarily the same as the expected emotion. For the present study, each participant performed 100 trials of emotion induction using pictures (one picture per trial) selected from the IAPS database as the emotional stimuli, with 25 trials for each of the four emotional categories in the valence-arousal space. The distribution of the selected pictures in the valance-arousal level plane is shown in Figure 3.

#### 4.1.3. Emotion Induction Experimental Paradigm and EEG Labeling

**Figure 4.**
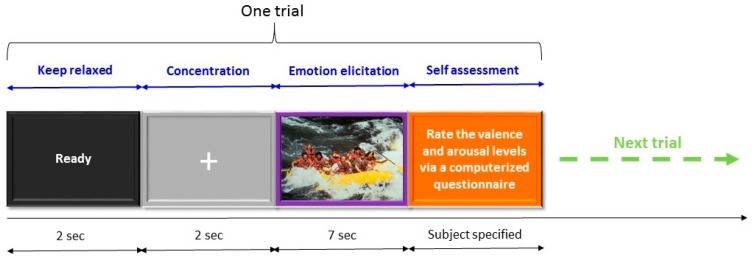
Emotion induction paradigm designed used for collecting EEG data.

The emotion induction procedure used in this study is illustrated in Figure 4. Each trial started with a 2-s ready cue followed by a 2-s period during which participants were asked to focus on the fixation cross centered at the computer monitor with a gray-level background and try not to think anything on purpose. Subsequently, there was a 7-s emotion induction period during which one of the selected 100 IAPS pictures was displayed on the PC screen and participants were instructed to try to engage themselves into the emotion that the picture may represent. At the end of each trial, participants took a short break to assess their subjective emotional experience during the emotion-induction period in the trial using a computerized questionnaire. Note that the current experiment was a self-paced design in which participants could initiate the next trial, and they were also allowed to decide when to take a longer break. The 7-s EEG signal acquired during the emotion induction period was labeled with the self-assessment result. Namely, for the 7-s EEG signal, its arousal level was labeled as either high or low and its valence was also labeled as either high or low. The labeled EEG signal was further partitioned into two 3.5-s EEG epochs. In other words, two EEG epochs having the same valence and arousal labels were collected for each trial. Finally, participants were instructed to press any key on a keyboard to start the next trial in which a different IAPS picture was presented during the emotion induction period.

We used a block design in which four emotional categories were blocked into four runs. Within each run, however, the pictures were randomly presented to prevent the order effects, as what the Reviewer said. The reason to use a block design was to increase the emotional stability within a block and reduce the possibilities of potential “contamination” from left-over emotional experience from the previous trial.

After the entire emotion-induction experimental procedure consisting of 100 trials was performed, we collected 200 EEG epochs of 3.5-s (hereafter called EEG data) from each participant. The EEG data labeling results were summarized in Table 1. It is important to note that even though the numbers of stimuli with high-arousal/valence and low-arousal/valence based on the IAPS rating were designed to be equal, the labeled EEG datasets based on the self-assessment procedure are imbalanced in all participants except for the tenth participant where the numbers of high-valence and low-valence EEG data are all equal to 100. Moreover, the EEG datasets are highly imbalanced in some participants. Take the first participant as an example. High arousal EEG data largely outnumber low arousal ones, resulting in a very imbalanced EEG data set: the imbalanced ratio is 146/54 = 2.70.

### 4.2. Results and Discussion

#### 4.2.1. Setting

Since the proposed feature extraction method KFEP is based on the multi-channel EEG spectral powers, we conducted a set of experiments to compare the proposed feature extraction method KFEP (*i.e.*, SP + KFDA) with other power spectrum based methods including EEG spectral powers (SP), the combination of EEG spectral powers and LDA (SP + LDA), the combination of EEG spectral powers and PCA (SP + PCA), and the combination of EEG spectral powers and kernel PCA (*i.e.*, the KEEP [27]). Here, the EEG spectral powers refer to the features generated by Layer 1 of the three-layer EEG-ER scheme (see Section 3.1 and Figure 2). For the method KFEP, its free parameters include the number of projection vectors and the width of the Gaussian σ. The KEEP method also has two free parameters, including the number of eigenvectors of the mapped-data covariance matrix and the kernel parameter σ. As for the classifiers, we compared the IQK-SVM (as well as imbalanced SVM, I-SVM) with traditional SVM since it was proposed to deal with the SVM's problem in EEG-ER. Notice that I-SVM is obtained by the first training step of IQK-SVM. The simple classifier *k*-NN (*k* = 3 in this study) was used as a baseline performance measurement for evaluating the improvements of the classification accuracy with the more sophisticated emotion classifiers SVM and IQK-SVM. The features and the emotion classifier to be compared in the experiments are summarized in Table 2.

#### 4.2.2. Parameter Optimization

IQK-SVM has three parameters, σ, positive penalty weight *C*^+^,and negative penalty weight *C*^−^. Too many free parameters would result in a time-consuming parameter optimization process. We set up a reduced searching rule for the two penalty weights:

(20) $$\begin{array}{cc} C^{+}=\frac{\#N}{\#P+\#N}\times C, & C^{-}=\frac{\#P}{\#P+\#N}\times C \end{array}$$

where #*P* and #*N* denotes the numbers of positive and negative training data, respectively. The advantage of using this setting is two-fold. First, the smaller class (the class with fewer training data) is assigned with a heavier penalty, thereby avoiding the class-boundary-skew problem caused by an imbalanced training dataset. Second, this setting also allows a reduction of the parameter number of IQK-SVM from 3 (σ,*C*^+^,*C*^−^) to 2 (σ,*C*), thus being able to reduce the complexity of optimum parameter searching.

Parameters of each method were optimized using 2-fold cross validation of 10 independent runs. The two fold validation method was used to compensate the potential small EEG data sets for some participants: a 5-fold or 10-fold method would result in a very small test set size. For instance, Participant 1 has only 54 low-arousal EEG data (see Table 1), and therefore the number of negative test data is less than 11 if a 5-fold cross validation is performed. This implies that an incorrectly classified negative test data results in a considerably-large false positive rate, about 9%–10%. The criterion of the optimal parameter selection for each method was based on the classification error rate: the best parameters gave the lowest average error rate over the ten runs. However, since the EEG datasets acquired from the 10 participants are all imbalanced (except for the valence case of Participant 10), the usual error rate estimation:

(21) $$\frac{\#FN+\#FP}{\#TP+\#FN+\#TN+\#FP}$$

is not an appropriate performance measure, where #*TP* and #*FN* denotes the number of true positives and the number of false negatives, respectively. In contrast, the balanced loss defined as:

(22) $$BL=1-\frac{\text{TPR}+\text{TNR}}{2},\text{where TPR}=\frac{\#TP}{\#TP+\#FN}\text{and}\;\text{TNR}=\frac{\#TN}{\#TN+\#FP},$$

is more appropriate [50,59]. We therefore use the balanced loss as the error rate estimation for the current study. Furthermore, the results reported in the following figures and tables are the best 10-run two-fold cross validation accuracies, *i.e.*, the lowest average error rates.

#### 4.2.3. Recognition Results and Comparision

**Figure 5.**
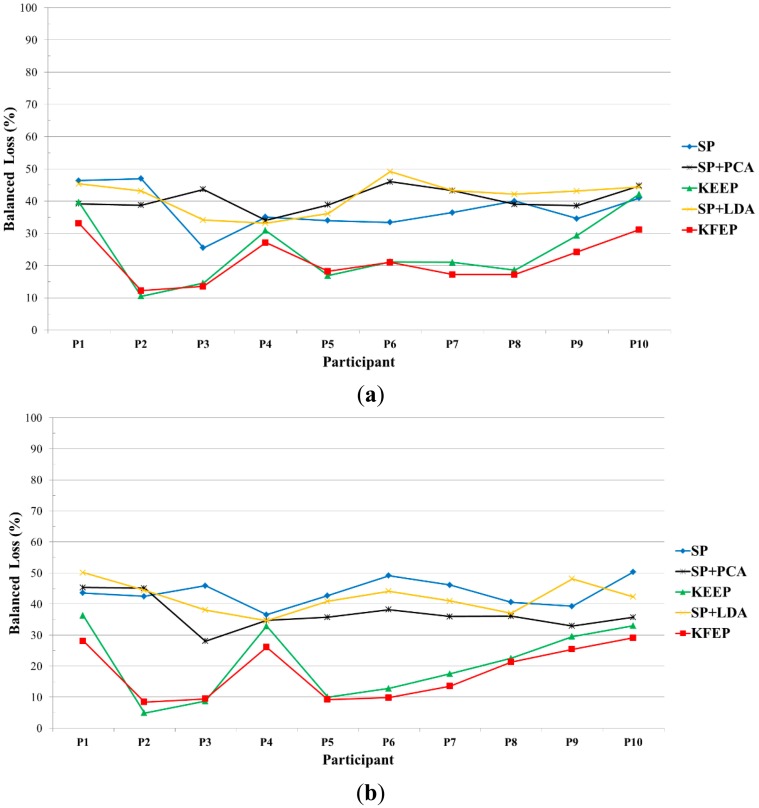
Comparison of the 10-participant error rates among different feature extraction methods in (**a**) valence classification and (**b**) arousal classification, where the classifier is *k*-NN method.

The error rates of the 10 participants for different feature extractions using *k*-NN classifier are shown in Figure 5. We can see from this figure that valence (shown in Figure 5a) or arousal (shown in Figure 5b) classification accuracies vary with participant. For instance, using the KFEP as feature, the error rate of Participant 2 is 12% while the one of participant 1 is above 30% in terms of valence classification. Moreover, SP + PCA is not always a better feature than SP, which indicates that using PCA to further extract second-order corrections from the SP features does not guarantee a better result. It is worth to note that SP + LDA performs much worse than SP in most cases and possible explanation is discussed as follows.

**Figure 6.**
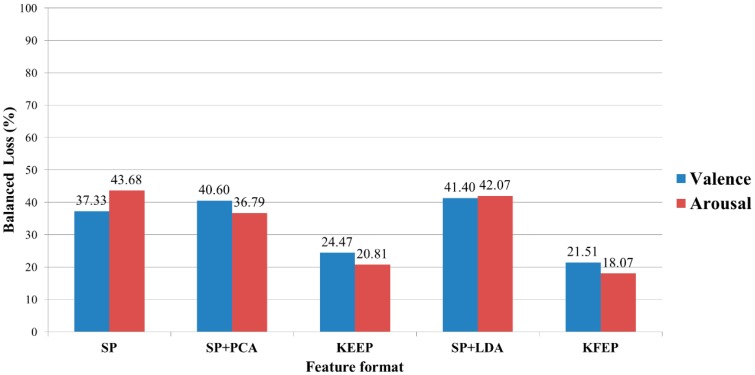
Average error rates of the 10 participants for different features using *k*-NN classifier.

Looking back at Figure 2, we can see that the SP vector **z** consists of 5 × *N_c_* spectral power values. In this study, the number of electrodes is 62, and therefore the dimension of the SP vector **z** is 310. Moreover, the numbers of training data for all the participants are smaller than 310. For instance, participant 1 has 76 high-valence data and 124 low-valence data. When performing the 2-fold cross validation, the training set has only 100 data (38 are positive and 62 are negative). Clearly, the number of training data is much smaller than the dimension of SP vector. In other words, LDA actually suffered from the so-called *small sample size* (SSS) problem [60] in our study, and therefore the performance of LDA dropped significantly. Furthermore, as shown in Figure 6, the proposed feature extraction method KFEP achieves the best results in both valence (21.51%) and arousal (18.07%) classifications, and performs much better than the traditional EEG spectral power features (37.33% and 43.68%). This comparison indicates the validity of using KFDA method to improve the discriminability of the widely-used EEG spectral powers in EEG-ER.

After comparing the classification performances of these features by *k*-NN classifier, the classification accuracies of other classifiers (SVM, I-SVM, and IQK-SVM) were further tested. The average error rates of the 10 participants are listed in Table 3. Note that both SP + PCA and SP + LDA are no longer involved in following comparison because the two perform much worse than KEEP and KFEP when the *k*-NN is used as the classifier (Figure 6).

It can be observed from Table 3 that when the classifier is fixed, the feature KEEP outperforms the other two. For example, when SVM is used, KFEP outperforms SP and KEEP by 11.6% (31.84%–20.18%) and 7.25% (27.43%–20.18%) in valence classification, respectively. Moreover, when the feature extraction method is fixed, the sophisticated classifier SVM is not consistently better than the simple *k*-NN classifier. For example, when the chosen feature is SP, *k*-NN (43.68%) is even slightly better than SVM (44.79%) in the case of arousal classification. The same situation occurs in the case where the KEEP is adopted as the feature. In that case, the error rate of SVM is 25.54%, which is much larger than that (20.81%) of *k*-NN classifier.

By further comparing SVM with I-SVM, we can see that I-SVM outperforms SVM in all cases, especially in the case of arousal classification. For example, when SP is used as the feature, I-SVM outperforms SVM by 12.51% (44.79%–32.28%) in arousal classification: the performance difference between SVM and I-SVM is considerably large. A possible reason is that the positive and negative data for all participants are very imbalanced (see Table 1); thus the effect of using DEC algorithm to improve SVM's classification accuracy is significant (considering that I-SVM is essentially a DEC algorithm-based SVM). Interestingly, when KFEP is the feature, the difference between SVM and I-SVM becomes small instead in arousal classification (18.21% – 17.23% = 0.98%). This could be due to the fact that when using the KFEP as the feature, the error rate becomes relatively smaller compared with using SP as the feature, implying that the overlap region between positive (high arousal) and negative (low arousal) classes becomes relatively small in the space of EEG patterns. Since the overlap region between the two classes is small, the number of training errors becomes small too. As a result, the class-boundary-skew phenomenon of the learned OSH is not significant even though the two classes are very imbalanced. The effect of using the DEC algorithm to improve SVM's classification accuracy is thus limited. In other words, there is no need to use DEC algorithm to improve SVM's performance when error rate is small.

Finally, we can see from Table 6 that IQK-SVM performs the best in all cases. The best valence and arousal classification results are achieved when KFEP feature and IQK-SVM classifier are adopted: the error rates for the two classification tasks are only 17.32% and 15.21%, respectively. Compared with the results of previous single-trial EEG-ER systems (See Figure 1), our proposed EEG-ER scheme achieves the highest EEG-ER accuracies: 82.68% for valence classification and 84.79% for arousal classification. However, in practice, such comparison is unfair, because the experimental settings are different in terms of the number of electrodes in use, types of emotional stimulus, and participants. Nevertheless, our results demonstrate that the discriminability of traditional EEG spectral power features (powers of theta, alpha, beta, gamma bands) can much improved by applying the KFDA method to these spectral powers (*i.e.*, the proposed feature KFEP) and the proposed IQK-SVM is a more powerful classifier for the valence and arousal classification.

#### 4.2.4. Comparison between the First and the Second 3.5-s EEG Signals

To analyze the homogeneity of the EEG signals in the 7-s intervals and compare the consistency between the first and second 3.5-s EEG epochs, we implemented the following two experiments:

(1) Experiment 1 (first 3.5-s): We test the classification accuracies of valance and arousal of only the first 3.5-s EEG epochs with a ten-run two-fold cross validation procedure.
(2) Experiment 2 (second 3.5-s). We test the classification accuracies of valance and arousal of only the second 3.5-s EEG epochs with a ten-run two-fold cross validation procedure.

**Figure 7.**
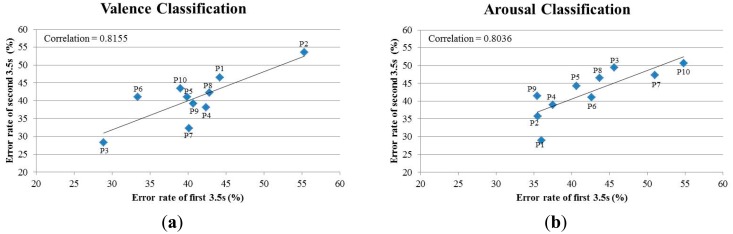
Scatter plots of the error rates of the first and second 3.5-s for (**a**) valence and (**b**) arousal classifications using SP feature and *k*-NN classifier.

**Figure 8.**
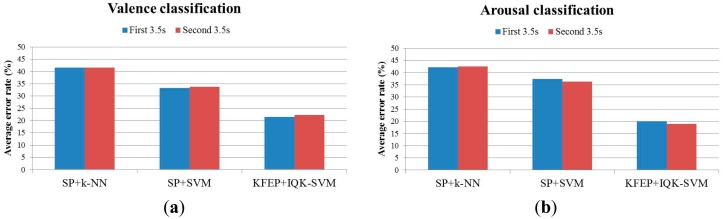
Comparison of average error rates between the first and second 3.5-s for different methods: (**a**) valence and (**b**) arousal classifications.

The testing rationale is that if the signals in the 7-s intervals have a high homogeneity, the first and second 3.5-s EEG epochs will result in similar classification accuracies. In this experiment, we used spectral powers (SP) as features and *k*-NN as the classifier. Our results, as listed in Table 4, show that the classification accuracies of the first and second 3.5-s EEG epochs are in general very similar to each other. The average error rates (N = 10) of the first 3.5-s and second 3.5-s are 41.56% and 41.54% respectively for the valence classification and 42.28% and 42.48% respectively for the arousal classification. The similar classification accuracies of the first 3.5-s and second 3.5-s EEG epochs indicate a high homogeneity/consistency of the signals in the 7-s intervals. Furthermore, as revealed in the scatter plots of the classification error rates in Figure 7, there is a high correlation between the classification performance of the two 3.5-s intervals for both the valence (*r* = 0.8036) and arousal (*r* = 0.8155) classifications. We also compare the classification performance between different classification methods (Figure 8), and again, the two 3.5-s intervals produce almost the same classification accuracy for each of the methods, with the proposed method (KFEP + IQK-SVM) provides the lowest average error rates.

#### 4.2.5. Reliability Test

To test the reliability of the emotion-induction experiment, we apply a split half procedure to the EEG data set. We split the data set of each participant into two disjoint subsets (subset A and subset B), and each subset contains the EEG data recorded from 50 trials (*i.e.*, the EEG induced from 50 different IAPS pictures). We test the valence and arousal classification accuracies on each subset independently by performing a leave-one-out cross validation on each subset. The results listed in Table 5 show that the classification performances of the two subsets are highly correlated for both the valence (*r* = 0.7711) and arousal (*r* = 0.8520) classifications, suggesting that the emotion-induction procedure has a high reliability. These findings also reveal that it will be possible to design an EEG-ER system that uses less emotional stimuli for classifier training.

#### 4.2.6. Comparison of Classification Accuracies between IAPS Rating and Self-Assessment Labeling

For each participant, a ten-run two-fold cross validation procedure is performed on the EEG epochs labeled based on the IAPS ratings. The average error rate (*i.e.*, average balanced loss) over the ten participants are listed in Table 6. Note that the results based on the self-assessment labeling are identical to the ones listed in Table 3.

We can observe from Table 6 that when SP is the feature and *k*-NN is the classifier, the average error rate based on the IAPS rating (43.23%) is much higher than that based on the self-assessment labeling (37.33%) for the valence classification while the average error rates for the two labeling method are very similar (IAPS rating: 43.60%, self-assessment: 43.68%) for the arousal classification. When we use SVM as the classifier, the difference between the results based on the two labeling methods become more prominent for the arousal classification (IAPS rating: 38.88%, self-assessment: 44.79%). Moreover, by comparing SP + *k*-NN with SP + SVM in the case of arousal classification, the IAPS-rating result is improved (from 43.60% to 38.88%) while the self-assessment result becomes slightly worse (from 43.68% to 44.79%). This could be due to fact that the EEG dataset labeled by IAPS rating is balanced while the dataset labeled by self-assessment is highly imbalanced (see Table 1): when SVM is trained with imbalanced dataset, its generalizability becomes worse. Regardless of the data labeling method, however, our proposed method (KFEP + IQK-SVM) still performs the best among all testing methods. Overall, the self-assessment labeling achieves higher classification accuracies of valence and arousal. Therefore, we can conclude here that the self-assessment procedure is more appropriate for establishing an EEG-based emotion recognition system.

## 5. Conclusions

In this paper, we have developed a three-layer EEG-ER scheme for solving two crucial classification problems: valence and arousal classifications. In this scheme, we adopted the commonly used EEG spectral powers as the basis and further proposed a novel feature extraction method KFEP. Results have shown that using KFEP as the emotional EEG features can result in better classification accuracy than the EEG spectral powers. Moreover, we also propose a robust emotion classifier called IQK-SVM by introducing the DEC algorithm and the quasiconformal kernel transformation into the traditional SVM. The results have also indicated that IQK-SVM can perform better than SVM in both arousal and valence classifications.

The intent of this work is to improve widely used methods in previous EEG-ER systems, including spectral-power features and SVM classifier. Although the results have demonstrated the effectiveness of the proposed methods, it is believed that the currently obtained EEG-ER accuracy can be further improved by optimizing other factors that are also crucial in EEG-ER, such as finding the most emotion-informative electrodes [17], using an emotion induction index to collect more representative EEG data [32]. Moreover, a very recent study has suggested that the regular EEG frequency bands are not necessarily the best for EEG-ER [28]: some sub-bands are more useful instead. Therefore, the method of filter bank spectral powers could achieve better EEG-ER accuracy than the regular spectral power bands (e.g., theta, alpha, low beta, high beta, and gamma bands). Another interesting future research direction is to design an EEG-ER system based on a three-dimensional model [61] of emotion (*i.e.*, valence, arousal, dominance) instead of a two-dimensional model. An emotion model with higher dimensionality will presumably be able to support finer categorization of emotional states.

## Figures and Tables

**Table 1.** EEG data labeling results for the ten participants.

**Participants**	**Valence**			**Arousal**		

	**High (Positive Class)**	**Low (Negative Class)**	**Imbalanced Ratio (High/Low)**	**High (Positive Class)**	**Low (Negative Class)**	**Imbalanced Ratio (High/Low)**
1	76	124	0.61	146	54	2.70
2	108	92	1.17	136	64	2.13
3	114	86	1.33	114	86	1.33
4	96	104	0.92	116	84	1.38
5	112	88	1.27	132	68	1.94
6	102	98	1.04	134	66	2.03
7	86	114	0.75	114	86	1.33
8	104	96	1.08	126	74	1.70
9	112	88	1.27	132	68	1.94
10	100	100	1	94	106	0.89

	Average imbalanced ratio		1.04	Average imbalanced ratio		1.73

**Table 2.** Features and the emotion classifier to be compared in the experiments.

**Feature Extraction Methods**	**Emotion Classifiers**
SP, SP + PCA, SP + LDA, KEEP (SP + kernel PCA), KFEP (SP + KFDA)	*k*-NN, SVM, I-SVM, IQK-SVM

**Table 3.** Average error rates of 10 participants for different combinations of feature and classifier (%).

	**SP**		**KEEP**		**KFEP**	

	**VC**	**AC**	**VC**	**AC**	**VC**	**AC**
***k*-NN**	37.33	43.68	24.47	20.81	21.51	18.54
**SVM**	31.84	44.79	27.43	25.54	20.18	18.21
**I-SVM**	28.43	32.28	24.87	23.03	18.28	17.23
**IQK-SVM**	24.90	28.32	21.93	19.87	17.32	15.21

VC: valence classification; AC: arousal classification.

**Table 4.** Average error rates of 10 participants for the first and second 3.5-s EEG intervals (%) using SP feature and *k*-NN classifier.

	**Valence Classification**		**Arousal Classification**	
	
	**First 3.5 s**	**Second 3.5 s**	**First 3.5 s**	**Second 3.5 s**
**Participant 1**	44.20	46.50	36.02	28.88
**Participant 2**	55.30	53.50	35.50	35.70
**Participant 3**	28.88	28.27	45.61	49.39
**Participant 4**	42.40	38.10	37.50	38.90
**Participant 5**	39.90	41.00	40.60	44.30
**Participant 6**	33.37	41.02	42.65	41.02
**Participant 7**	40.10	32.24	51.02	47.35
**Participant 8**	42.80	42.20	43.67	46.53
**Participant 9**	40.70	39.20	35.40	41.40
**Participant 10**	39.00	43.40	54.80	50.61
**Ave. error rate**	**41.56**	**41.54**	**42.28**	**42.48**

**Table 5.** Average error rates of the split half procedure (%) using SP feature and *k*-NN classifier.

	**Valence Classification**		**Arousal Classification**	

	**Subset A**	**Subset B**	**Subset A**	**Subset B**
**Participant 1**	42	44	33	30
**Participant 2**	46	41	38	39
**Participant 3**	28	25	46	50
**Participant 4**	31	35	40	41
**Participant 5**	36	31	42	43
**Participant 6**	42	47	42	38
**Participant 7**	41	36	43	46
**Participant 8**	43	39	43	47
**Participant 9**	37	39	34	39
**Participant 10**	40	44	50	48
**Ave. error rate**	**38.60**	38.10	**41.10**	**42.00**
**Correlation Coe.**	**0.7711**		**0.8520**	

**Table 6.** Comparison of average error rates (in %) between the classification accuracy based on the IAPS-rating and the self-assessment labeling.

	**Valence Classification**		**Arousal Classification**	

	**IAPS Rating**	**Self-Assessment**	**IAPS Rating**	**Self-Assessment**
SP + *k*-NN	43.23	37.33	43.60	43.68
SP + SVM	37.12	31.84	38.88	44.79
KFEP + IQK-SVM	28.34	17.32	29.23	15.21
